# Enhancing Pure Inertial Navigation Accuracy through a Redundant High-Precision Accelerometer-Based Method Utilizing Neural Networks

**DOI:** 10.3390/s24082566

**Published:** 2024-04-17

**Authors:** Qinyuan He, Huapeng Yu, Dalei Liang, Xiaozhuo Yang

**Affiliations:** 1 National Innovation Institute of Defense Technology, Academy of Military Science, Beijing 100071, China; 15285550382@163.com (Q.H.); liangdalei1114@163.com (D.L.); 2 School of Automation Engineering, University of Electronic Science and Technology of China, Chengdu 611731, China; 202222060218@std.uestc.edu.cn

**Keywords:** inertial navigation, deep learning, redundant accelerometer

## Abstract

The pure inertial navigation system, crucial for autonomous navigation in GPS-denied environments, faces challenges of error accumulation over time, impacting its effectiveness for prolonged missions. Traditional methods to enhance accuracy have focused on improving instrumentation and algorithms but face limitations due to complexity and costs. This study introduces a novel device-level redundant inertial navigation framework using high-precision accelerometers combined with a neural network-based method to refine navigation accuracy. Experimental validation confirms that this integration significantly boosts navigational precision, outperforming conventional system-level redundancy approaches. The proposed method utilizes the advanced capabilities of high-precision accelerometers and deep learning to achieve superior predictive accuracy and error reduction. This research paves the way for the future integration of cutting-edge technologies like high-precision optomechanical and atom interferometer accelerometers, offering new directions for advanced inertial navigation systems and enhancing their application scope in challenging environments.

## 1. Introduction

Inertial navigation technology is a crucial technique for various vehicular platforms to execute tasks and enhance stealth. However, due to the continuous accumulation of inertial navigation errors over time, the demand for high-precision inertial navigation for extended missions remains unmet. There are mainly two methods to improve the reliability and accuracy of the inertial navigation system (INS): one is to enhance the reliability and precision of individual instruments, which imposes higher requirements on the manufacturing processes and technical specifications of inertial instruments, increasing research costs and implementation challenges; the other method involves adopting redundancy schemes to enhance the system’s reliability and accuracy, which is a more cost-effective and easier-to-implement approach.

Redundant inertial navigation technology encompasses the following three forms: analytical redundancy, software redundancy, and hardware redundancy. Analytical redundancy, based on the knowledge of rotational and translational dynamics, is a method that can improve hardware redundancy performance [1]. It generates additional redundant observational data to enhance the system’s fault diagnosis capability without improving the navigation system’s accuracy and is mostly applied in fault detection. Software redundancy uses multiple algorithms to enhance the reliability of inertial navigation solutions, preventing errors due to software design and computational faults, but it does not directly enhance the precision of inertial navigation solutions. Hardware redundancy, on the other hand, leverages multiple inertial navigation systems and sensors to enhance the overall system performance. In this configuration, redundant sensor systems operate independently while cooperating during the operation, integrating various measurement data through data fusion algorithms to achieve superior performance compared to a single INS, which has been widely applied in aviation [2] and aerospace [3] fields. Hardware redundancy technology can be divided into system-level redundancy and device-level redundancy [4]. System-level redundancy, composed of two or more INS sets, processes output data from each subsystem collectively to improve the INS’s navigational performance, significantly enhancing system reliability but also increasing system complexity and costs, mainly applied in manned spacecraft and aviation sectors with high safety and extended operation time requirements. Device-level redundancy involves redundant design at the instrument level, mainly suitable for flexibly structured strapdown inertial navigation systems, offering significant advantages in weight, volume, and cost, and is ideal for widespread adoption. Among these, inertial accelerometers, often less costly than gyroscopes within the INS, provide a cost-effective redundancy option. Currently, accelerometer types include pendulous integrating gyroscopic accelerometers, flexural accelerometers, quartz-tuning fork accelerometers, silicon micromechanical accelerometers, micro-optical accelerometers, atomic accelerometers, and optomechanical accelerometers, and especially the latter few are in the exploratory stages of laboratory development or initial application, promising broad future prospects.

Addressing redundancy configuration in inertial navigation, different sensor quantities, and geometric arrangements significantly impact system performance and reliability. In the 1970s, Evans et al. [5] proposed a dodecahedral redundancy structure with six gyroscopes and six accelerometers. In the early 1980s, Boeing [6] introduced a redundant inertial device comprising five accelerometers and five gyroscopes. Gilmore et al. [7] proposed an inertial navigation structure with a dodecahedral redundancy configuration. Pejsa et al. [8] provided optimal schemes for four, five, and six sensor redundancy configurations. Research by Abdallah Osman et al. [9] explored the impact of skewed redundant inertial measurement units (IMUs) on redundant INS performance, demonstrating that increased sensor redundancy significantly enhances system performance. Duk-Sun Shim et al. [10] developed a method to determine the optimal navigation combination and fault detection and isolation (FDI) performance.

With traditional inertial navigation methods approaching developmental thresholds, the integration of neural networks with inertial navigation has recently become a focal point of research. Chang et al. [11] proposed a method using Long Short-Term Memory Recurrent Neural Networks (LSTM-RNNs) for denoising Microelectromechanical Systems (MEMSs) IMU. Martin Brossard et al. [12] introduced a learning method for denoising IMU gyroscopes using real-world data. Esfahani et al. [13] presented AbolDeepIO, a deep neural network with three channels, extracting and learning training quantities from tri-axial accelerometer and gyroscope information, including the sampling interval between two IMUs, to aid feature extraction and training. Topini et al. [14] proposed an LSTM-based Dead Reckoning (DR) method to estimate pitch and roll rates. They used end-to-end navigation methods with unidirectional and bidirectional LSTM to process raw sensor data and then used the output of LSTM and the time interval of the aforementioned cycle to obtain the position of the AUV. Song et al. proposed NN-DR [15], which uses neural networks to explore the time-varying relationship between acceleration and pitch angle using a single accelerometer. Research by Bai et al. [16] introduced a novel approach for location estimation using feature mode matching with deep network models, demonstrating that aligning specific deep learning models with categorized motion features significantly enhances the accuracy of pedestrian location estimation in environments where GPS is unavailable. Research by Li et al. [17] unveiled a cutting-edge hybrid algorithm that merges a Gated Recurrent Unit (GRU) neural network with an interactive multiple model adaptive robust cubature Kalman filter (IMM-ARCKF) aimed at refining the accuracy of the INS/GPS-integrated navigation system amidst GPS disruptions.

However, research on integrating device-level redundant inertial navigation systems with neural networks is still in its infancy. Neural networks, with their strong nonlinear fitting capabilities, can delve deeper into the additional information introduced by extra devices to enhance navigation precision.

The remainder of this paper is organized as follows: Section 2 provides a brief introduction to the background of inertial navigation and redundancy methods and perspectives. Section 3 describes the architecture and workflow of the displacement error prediction network. Section 4 introduces experiments based on actual sea trials and discusses the results in conjunction with real-world positioning outcomes. Section 5 concludes the paper and offers a perspective on future applications. This research proposes a navigation system structure based on redundant high-precision accelerometers without altering the existing INS framework. The research introduces a redundant high-precision accelerometer-based navigation system structure without altering the existing INS framework, designing a displacement error prediction network based on this structure and leveraging neural networks to delve into the positioning information provided by high-precision accelerometers. The main contributions include the following:

1. Proposing an easily implementable redundant high-precision accelerometer-based navigation system structure in conjunction with the current state of high-precision accelerometers, updating the Kalman filter error observation equation based on additional high-precision accelerometers.
2. Exploiting the time-varying error characteristics of the INS and redundant high-precision accelerometers to extract additional information, designing a targeted displacement error prediction network.
3. Combining theoretical design with practical platform construction and validating the proposed method through sea trials, demonstrating its effectiveness.

## 2. Background

### 2.1. Navigation Coordinate System

The navigation frame *n* is selected as the East–North–Sky (ENS) coordinate system, with the heading angle denoted as φ, the roll angle as γ, and the pitch angle as θ. Rotations around the three coordinate axes correspond to three independent direction cosine matrices, denoted as $C_{z}\left(\varphi \right)$, $C_{x}\left(\theta \right)$, and $C_{y}\left(\gamma \right)$. The attitude transformation matrix from the navigation frame *n* to the body frame *b*, denoted as $C_{n}^{b}$, and the transformation matrix from the body frame *b* to the navigation frame *n*, denoted as $C_{b}^{n}$, is expressed by the following equations:

(1) $$C_{n}^{b}=C_{y}(\gamma )C_{x}(\theta )C_{z}(\varphi )$$

(2) $$C_{b}^{n}=(C_{n}^{b}{)}^{T}=C_{z}{\left(\varphi \right)}^{T}C_{x}{\left(\theta \right)}^{T}C_{y}{\left(\gamma \right)}^{T}$$

We can assume that the acceleration sensed by the accelerometer related to the body is as follows:

(3) $$f^{b}=\left[\begin{array}{c} f_{x}^{b}\\ f_{y}^{b}\\ f_{z}^{b} \end{array}\right]$$

During navigation computation, it is necessary to transform $f^{b}$ to the navigation coordinate system to obtain $f^{n}$, which can be represented as shown below:

(4) $$f^{n}=C_{b}^{n}f^{b}$$

To acquire the real-time $f^{n}$, it is essential to update the transformation matrix $C_{b}^{n}$ based on the output of the gyroscope.

(5) $$C_{b}^{n}=C_{b}^{n}\Omega_{nb}^{b}$$

The angular velocity of frame *b* relative to frame *n* is denoted as $\omega_{nb}^{b}$, with $\Omega_{nb}^{b}$ representing the skew-symmetric matrix of the angular velocity $\omega_{nb}^{b}$. By utilizing the real-time-updated $C_{b}^{n}$, the vehicle’s attitude angles can be computed. Utilizing the accelerometer’s output acceleration data $f^{b}$ and the attitude transformation matrix $C_{b}^{n}$, the vehicle’s acceleration $a_{x}$ and $a_{y}$ in the navigation frame can be calculated. If the vehicle’s initial velocity is $v_{x0}$, $v_{y0}$ and the initial position is $L_{0}$, $J_{0}$, the vehicle’s velocity and position at time *t* can be computed as follows, assuming a constant rate of change in velocity and no external forces other than gravity affecting the linear acceleration:

(6) $$v_{x}=v_{0}+\int_{0}^{t}a_{x}dt$$

(7) $$v_{y}=v_{0}+\int_{0}^{t}a_{y}dt$$

(8) $$L=L_{0}+\frac{1}{R}\int_{0}^{t}v_{x}dt$$

(9) $$J=J_{0}+\frac{1}{R}\int_{0}^{t}\frac{1}{\cos L}v_{y}dt$$

### 2.2. Redundant Configuration Methods and Angles

The redundant configuration of inertial sensors can enhance the reliability and accuracy of inertial navigation systems. The redundancy in sensors fortifies the system’s capability to detect and isolate faulty sensors, thereby providing more accurate positional information.

**Figure 1:**
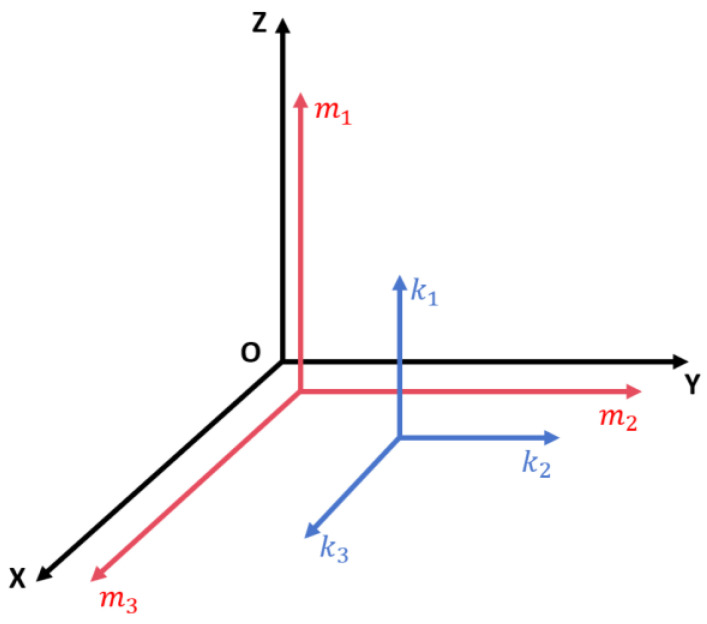
Accelerometer axis diagram.

This study contemplates a universally applicable redundancy scheme, acknowledging that accelerometers are often significantly less expensive than gyroscopes; Therefore, selecting these as high-precision components for redundancy strategies presents significant opportunities for their broader application and implementation. The redundant structures of inertial sensors within inertial navigation systems can be broadly classified into orthogonal and skewed configurations. A skewed configuration entails orienting the sensor’s sensitive axis at an angle to the coordinate axes, which, although it yields richer information in the sensor output, typically demands higher precision in the installation and incurs higher costs. This could introduce larger errors in installation and measurement. Consequently, this research opts for the more universally applicable orthogonal configuration approach. As illustrated in Figure 1, the high-precision accelerometer orthogonal configuration scheme employs “*m*” accelerometers from the original inertial navigation system and adds a group of “*k*” high-precision accelerometers. The axial orientation of the newly added high-precision accelerometers aligns with the original inertial navigation system’s coordinate axes. In such a configuration, the presence of two accelerometers measuring the acceleration along the same axis simplifies the system’s configuration equations, eases computation, minimizes measurement errors, and facilitates the detection of accelerometer faults.

Through the implementation of sensor redundancy, enhancements in system reliability and accuracy can be achieved. The research by Abdallah Osman et al. [9] substantiates that an increase in the quantity of redundant sensors significantly bolsters system performance. Nonetheless, given the considerations of cost and universality, this study employs a strategy of adding an extra set of tri-axial accelerometers, which elevates precision by an order of magnitude as the experimental scheme. Should the need arise, additional redundant sensing units can be integrated to further improve the system’s error detection and correction capabilities as well as its navigational precision.

### 2.3. Neural Network Algorithms

Neural networks are a category of intelligent algorithms used for effectively processing and categorizing data, and they are widely applied across various engineering fields. Their defining feature allows computers to learn and make corrections from network training and prior knowledge without explicit programming. These networks are extensively utilized in image recognition, voice input, text translation, and the automatic dissemination of news items, posts, or products that might interest users. Deep learning expands conventional neural networks into a large, scalable network architecture. Comprising multiple neural network layers, these deep learning algorithms can autonomously extract and learn relevant features from data. Consequently, more complex data processing applications can be mapped onto deep learning networks.

In recent years, the deep learning domain has notably focused on the Transformer architecture. Proposed by Google in 2017 [18], the Transformer model, characterized by its attention mechanism, which is not limited by local interactions, can effectively unravel long-range dependencies. This capability significantly impacted the field of natural language processing, offering superior performance in translation and recognized as a milestone model following recurrent neural network (RNN) and convolutional neural network (CNN), with contemporary large-scale models like Generative Pre-Trained Transformers (GPTs) based on it. As research progressed, the Transformer also found extensive application in image processing. Utilizing an encoder–decoder structure, the Transformer incorporates positional encoding to add and store positional information for each input token. Both the encoder and decoder in the Transformer apply multi-head attention, using scaled dot-product attention as its core component to provide a global focus and prevent gradient vanishing issues. In this paper, considering its excellent capability in handling context-based data and combined with the error characteristics of inertial navigation systems and high-precision accelerometers, the Transformer is chosen as the architecture for the displacement error prediction network.

## 3. Our Approach

**Figure 2:**
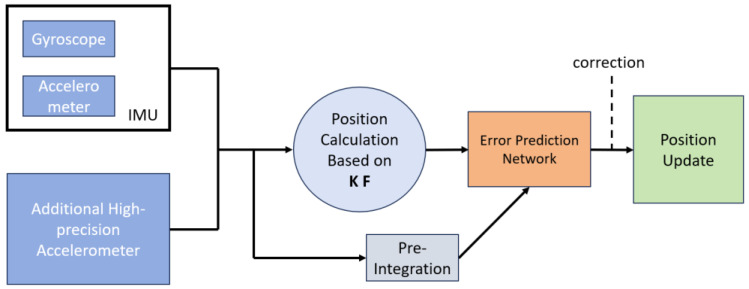
Redundant high-precision accelerometer inertial navigation system.

The design flowchart for the redundant high-precision accelerometer-based inertial navigation system is depicted in Figure 2, with the workflow described as follows:

### 3.1. System Hardware Structure Design

**Figure 3:**
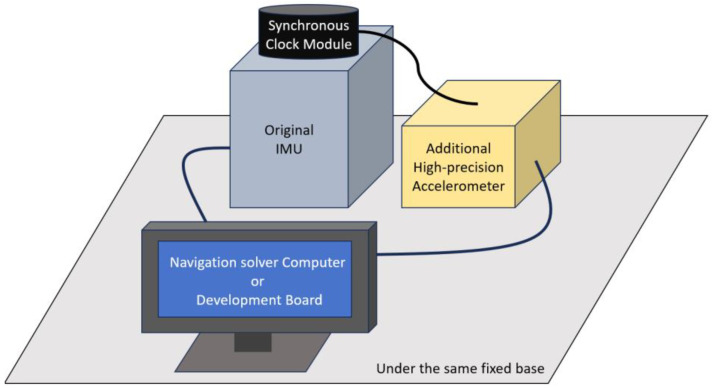
System hardware structure design diagram.

The hardware design of the entire system is illustrated in Figure 3. The main components include the existing inertial navigation system, an additional high-precision accelerometer group, a synchronization clock module, and a comprehensive navigation solution module. To enhance the universality and feasibility of the proposed method, the high-precision accelerometer group is configured orthogonally with the accelerometer group in the existing inertial navigation system. Within the inertial navigation system module, there are gyroscopes and “*m*” accelerometers, which provide data to the comprehensive navigation solution module. The “*k*” accelerometer group represents the high-precision accelerometers. The synchronization clock module is responsible for unifying control over the accelerometer information from both the high-precision accelerometer group and the existing inertial navigation system, sending synchronization clock signals and aligning frequencies and start points. 

The comprehensive navigation solution module encompasses a Position Calculation based on a Kalman filter and an error prediction network. Its inputs include the “*m*” accelerometer and gyroscope data from the inertial navigation system and the “*k*” accelerometer data from the redundant high-precision accelerometer module. The output from the existing inertial system and the high-precision accelerometer group is integrated using a Kalman filter to compute the positioning results. Then, using a neural network-based error prediction network, the system predicts and inverses the errors, outputting the low-frequency errors to the inertial navigation system’s updated algorithm for fusion with the positioning results. This process achieves the prediction and update of the position, ultimately outputting the navigation information after inverse correction and calculation.

### 3.2. The Multimode Switching for Motion States

The attitude error of SINS $\dot{\phi }$ can be expressed as follows:

(10) $$\dot{\phi }=\phi \times \omega_{in}^{n}+\delta \omega_{in}^{n}-\delta \omega_{ib}^{n}$$

where ϕ is the rotation vector from the ideal navigation coordinate system to the calculated navigation coordinate system, also called the misalignment angle error, $\omega_{in}^{n}$ represents the computed angular velocity values in the navigation frame, $\delta \omega_{in}^{n}$ represents the computed errors in the navigation frame, and $\delta \omega_{ib}^{n}$ represents the gyroscope measurement errors.

The gyroscope component error measurement model $\delta \omega_{ib}^{n}$ can be expressed as follows:

(11) $$\delta \omega_{ib}^{b}=\omega_{ibx}^{b}\delta K_{Gx}+\omega_{iby}^{b}\delta K_{Gy}+\omega_{ibz}^{b}\delta K_{Gz}+\varepsilon^{b}$$

where $\delta K_{G}$ is the scale factor and installation error matrix and $\varepsilon^{b}$ is the projection of gyro zero bias in the system.

The expressions of the rotation angular velocity $\omega_{ie}^{n}$ of the earth and the rotation angular velocity $\omega_{en}^{n}$ of the navigation system are

(12) $$\omega_{ie}^{n}=\left[\begin{array}{c} 0\\ \omega_{ie}\cos L\\ \omega_{ie}\sin L \end{array}\right],\omega_{en}^{n}=\left[\begin{array}{c} -v_{N}/(R_{M}+h)\\ v_{E}/(R_{N}+h)\\ v_{E}\tan L/(R_{N}+h) \end{array}\right]$$

The deviation of the above formula is obtained as shown below, respectively.

(13) $$\delta \omega_{ie}^{n}=\left[\begin{array}{c} 0\\ -\omega_{ie}\sin L\cdot \delta L\\ \omega_{ie}\cos L\cdot \delta L \end{array}\right]$$

(14) $$\delta \omega_{en}^{n}=\left[\begin{array}{c} -\delta v_{N}/R_{Mh}+v_{N}\delta h/R_{Mh}^{2}\\ \delta v_{E}/R_{Nh}-v_{E}\delta h/R_{Nh}^{2}\\ \tan L\cdot \delta v_{E}/R_{Nh}+v_{E}\sec^{2}L\cdot \delta L/R_{Nh}-v_{E}\tan L\cdot \delta h/R_{Nh}^{2} \end{array}\right]$$

where $\delta p={\left[\begin{array}{ccc} \delta L & \delta \lambda & \delta h \end{array}\right]}^{T}$ is the position error, δL, δλ, and δh represent the latitude, longitude and height errors, and $R_{M}$,$R_{N}$ represents the radius of curvature when the included angle between the normal section of the ellipsoid surface of the earth and the meridian plane is 0 and π/2, respectively.

We then substitute Equations (11), (13) and (14) into Equation (10) and rewrite the attitude equation as follows:

(15) $$\begin{array}{c} \;\dot{\phi }=\phi \times \omega_{in}^{n}+\delta \omega_{in}^{n}-\delta \omega_{ib}^{n}\\ \;\;\;\;=-(\omega_{in}^{n}\times )\phi +M_{2}\delta v^{n}+(M_{1}+M_{3})\delta p-\omega_{ibx}^{b}C_{b}^{n}\delta K_{Gx}-\omega_{iby}^{b}C_{b}^{n}\delta K_{Gy}-\omega_{ibz}^{b}C_{b}^{n}\delta K_{Gz}-C_{b}^{n}\varepsilon^{b} \end{array}$$

The speed error $\delta v^{n}$ is differentiated as follows:

(16) $$\begin{array}{c} \;\delta {\dot{v}}^{n}={\dot{\tilde{v}}}^{n}-{\dot{v}}^{n}\\ \;\;\;\;\;\;\;=({\tilde{C}}_{b}^{n}{\tilde{f}}_{sf}^{b}-C_{b}^{n}f_{sf}^{b})-\left[(2{\tilde{\omega }}_{ie}^{n}+{\tilde{\omega }}_{en}^{n})\times {\tilde{v}}^{n}-(2\omega_{ie}^{n}+\omega_{en}^{n})\times v^{n}\right]+({\tilde{g}}^{n}-g^{n}) \end{array}$$

where ${\tilde{f}}_{sf}^{b}=f_{sf}^{b}+\delta f_{sf}^{b}$, ${\tilde{\omega }}_{ie}^{n}=\omega_{ie}^{n}+\delta \omega_{ie}^{n}$, ${\tilde{\omega }}_{en}^{n}=\omega_{en}^{n}+\delta \omega_{en}^{n}$, ${\tilde{g}}^{n}=g^{n}+\delta g^{n}$, $\delta f_{sf}^{b}$ are the measurement errors of the accelerometer, respectively, and the angular velocity rotation error of the earth’s rotation, the rotation calculation error of the navigation system, and the gravity error are obtained by substituting them into Formula (12). To account for the accelerometer measurement errors, $\delta \omega_{ie}^{n},\delta \omega_{en}^{n},\delta g^{n},$ including the earth’s rotation rate rotation error, the navigation system rotation calculation error, and gravity error, ${\tilde{f}}_{sf}^{b},{\tilde{\omega }}_{ie}^{n},{\tilde{\omega }}_{en}^{n},{\tilde{g}}^{n}$ is substituted into Equation (12)

(17) $$\delta {\dot{v}}^{n}=f_{sf}^{n}\times \phi +v^{n}\times (2\delta \omega_{ie}^{n}+\delta \omega_{en}^{n})-(2\omega_{ie}^{n}+\omega_{en}^{n})\times \delta v^{n}+\delta f_{sf}^{n}+\delta g^{n}$$

The position error of the strapdown inertial navigation system (SINS) can be expressed as follows:

(18) $$\delta \dot{L}=\frac{1}{R_{M}+h}\delta v_{N}-\frac{v_{N}}{(R_{M}+h{)}^{2}}\delta h$$

(19) $$\delta \dot{\lambda }=\frac{\sec L}{R_{N}+h}\delta v_{E}+\frac{v_{E}\sec L\tan L}{R_{N}+h}\delta L-\frac{v_{E}\sec L}{(R_{N}+h{)}^{2}}\delta h$$

(20) $$\delta \dot{h}=\delta v_{U}$$

The velocity component of inertial navigation $v^{n}={\left[\begin{array}{ccc} v_{E} & v_{N} & v_{U} \end{array}\right]}^{T}$, and the velocity error component $\delta v^{n}={\left[\begin{array}{ccc} \delta v_{E} & \delta v_{N} & \delta v_{U} \end{array}\right]}^{T}$.

The measurement error model of the accelerometer module is as follows:

(21) $$\delta f_{sf}^{b}={\tilde{f}}_{sf}^{b}-f_{sf}^{b}\;\;\;\;\;\;\;\;\;\;\;\;\;=f_{sfx}^{b}\delta K_{Ax}+f_{sfy}^{b}\delta K_{Ay}+f_{sfz}^{b}\delta K_{Az}+\nabla^{b}\;$$

where $\delta K_{A}$ represents the matrix of the installation error and scale error, $f_{sf}^{b}$ and ${\tilde{f}}_{sf}^{b}$ represent the theoretical value of the specific force and $\nabla^{b}$ measures the output value of the accelerometer, respectively; zero bias for the accelerometer measurement is based on the inertial navigation system error models of Equations (15) and (16), and the system model can be obtained by expanding the gyro and table error models:

(22) $$\overset{•}{\left(\begin{array}{c} \delta L\\ \delta \lambda \\ \delta h\\ \delta V_{E}\\ \delta V_{N}\\ \delta V_{U}\\ \phi_{E}\\ \phi_{N}\\ \phi_{U}\\ \nabla_{x}\\ \nabla_{y}\\ \nabla_{z}\\ \varepsilon_{x}\\ \varepsilon_{y}\\ \;\;\;\varepsilon_{z} \end{array}\right)}=\left[\begin{array}{ccccc} \left(\begin{array}{ccc} 0 & -\omega_{enU}^{n} & -\omega_{enN}^{n}\\ \omega_{enU}^{n} & 0 & \omega_{enE}^{n}\\ \omega_{enN}^{n} & -\omega_{enE}^{n} & 0 \end{array}\right) & \left(\begin{array}{ccc} 1 & 0 & 0\\ 0 & 1 & 0\\ 0 & 0 & 1 \end{array}\right) & \left(0\right) & \left(0\right) & \left(0\right)\\ \left(0\right) & \left(\begin{array}{ccc} 0 & -\left(2\omega_{ieU}^{n}+\omega_{enU}^{n}\right) & -\left(2\omega_{ieN}^{n}+\omega_{enN}^{n}\right)\\ \left(2\omega_{ieU}^{n}+\omega_{ecU}^{n}\right) & 0 & \left(2\omega_{ieE}^{n}+\omega_{enE}^{n}\right)\\ \left(2\omega_{ieN}^{n}+\omega_{enN}^{n}\right) & -\left(2\omega_{ieE}^{n}+\omega_{enE}^{n}\right) & 0 \end{array}\right) & \left(\begin{array}{ccc} 0 & f_{U}^{n} & f_{N}^{n}\\ -f_{U}^{n} & 0 & -f_{E}^{n}\\ -f_{N}^{n} & f_{E}^{n} & 0 \end{array}\right) & C_{b}^{n} & \left(0\right)\\ \left(0\right) & \left(0\right) & \left(\begin{array}{ccc} 0 & -\omega_{inU}^{n} & -\omega_{icE}^{c}\\ \omega_{inU}^{n} & 0 & \omega_{inE}^{n}\\ \omega_{inN}^{n} & -\omega_{inE}^{n} & 0 \end{array}\right) & \left(0\right) & -C_{b}^{n}\\ \left(0\right) & \left(0\right) & \left(0\right) & \left(0\right) & \left(0\right)\\ \left(0\right) & \left(0\right) & \left(0\right) & \left(0\right) & \left(0\right) \end{array}\right]\left(\begin{array}{c} \delta L\\ \delta \lambda \\ \delta h\\ \delta V_{E}\\ \delta V_{N}\\ \delta V_{U}\\ \phi_{E}\\ \phi_{N}\\ \phi_{U}\\ \nabla_{x}\\ \nabla_{y}\\ \nabla_{z}\\ \varepsilon_{x}\\ \varepsilon_{y}\\ \;\;\;\varepsilon_{z} \end{array}\right)$$

In the process of integrated navigation, the speed measurement model is as follows:

(23) $$\Delta V={\tilde{V}}^{n}-V^{n}$$

where ${\tilde{V}}^{n}$ represents the velocity calculated by inertial navigation and $V^{n}$ represents the velocity calculated by redundant accelerometer module, where

(24) $${\tilde{V}}^{n}=V^{n}+\delta V^{n}$$

Equation (24) can be substituted into Equation (23) to obtain the measurement model of speed.

(25) $$\Delta V=\delta V^{n}$$

Equation (25) can be transformed considering the horizontal attitude observation at the same time, and then the overall observation equation Obs can be expressed as follows:

(26) $$X_{w}=\left(\delta L^{T}\delta \lambda^{T}\delta h^{T}\delta {V_{N}}^{T}\delta {V_{E}}^{T}\delta {V_{D}}^{T}{\phi_{N}}^{T}{\phi_{E}}^{T}{\phi_{D}}^{T}{\nabla_{x}}^{T}{\nabla_{y}}^{T}{\nabla_{z}}^{T}{\varepsilon_{x}}^{T}{\varepsilon_{y}}^{T}{\varepsilon_{z}}^{T}\right)$$

(27) $$Obs=\left[\begin{array}{ccccc} 0_{3\times 3} & \left(\begin{array}{ccc} 1 & 0 & 0\\ 0 & 1 & 0\\ 0 & 0 & 1 \end{array}\right) & \left(\begin{array}{ccc} 1 & 0 & 0\\ 0 & 1 & 0\\ 0 & 0 & 0 \end{array}\right) & 0_{3\times 3} & 0_{3\times 3} \end{array}\right]{X_{w}}^{T}$$

The Kalman filtering of integrated navigation based on the above system model and measurement model can realize the optimal integrated navigation of inertial navigation and accelerometer modules.

### 3.3. Design of Deep Learning Method for Low-Frequency Restoration of High-Frequency Errors

We acknowledge the well-established efficacy of Kalman filtering in providing optimal solutions for integrated navigation when system noise and characteristics are precisely modeled. Nevertheless, our manuscript introduces neural network augmentation for the Kalman filter, driven by our understanding of real-world operational scenarios where optimal Kalman filtering assumptions may not strictly apply. These scenarios include nonlinear system dynamics and non-Gaussian noise distributions. The integration of the neural network aims to complement, not replace, the Kalman filter. It leverages the neural network’s data-learning capability to adapt to unknown dynamics and disturbances, which is especially valuable in unanticipated or complex environments. Our approach utilizes the neural network’s strengths in pattern recognition and learning from extensive datasets to discern subtle patterns or trends from historical and real-time data.

**Figure 4:**
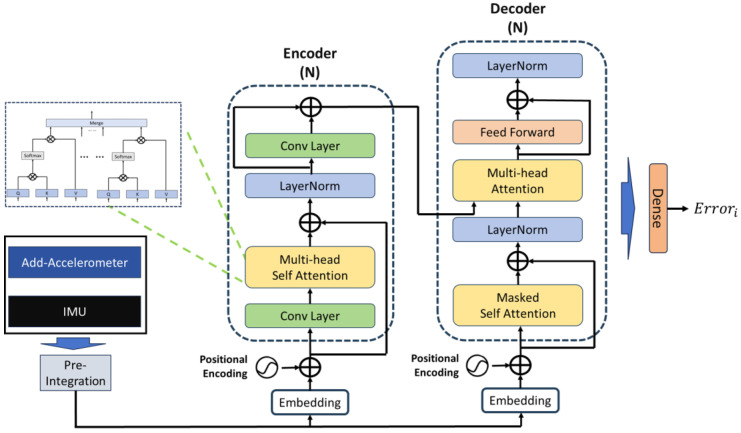
Displacement error prediction network structure diagram.

After integrating with the Kalman filter algorithm, our proposed algorithm leverages numerical data from high-precision accelerometers but has yet to fully exploit the precision data these redundant sensors offer. Therefore, this paper explores employing the deep learning neural networks’ nonlinear characteristics and learning capabilities to investigate the intrinsic attributes of high-precision accelerometers. Inertial positioning errors and attitude transformation errors are typically time-related. Traditional algorithms have not entirely eliminated these nonlinear time-varying errors and may even introduce and amplify existing ones. Thus, designing an error prediction network atop the current navigation algorithm to further mitigate errors and deeply mine the additional information provided by high-precision accelerometers is essential. The designed Displacement error prediction network structure is illustrated in Figure 4. This paper includes time information in the error prediction network’s computational input, enabling the neural network to generate time-adaptive nonlinear functions for improved error prediction. Compared to traditional models, our error prediction network incorporates time information as additional learning input, which is built around a Transformer-based network to fully leverage its sequential processing and context-aware learning capabilities. To prevent potential divergence in long-duration inertial navigation predictions due to overfitting time information, this study contemplates integrating time information into the network learning process, designing a real-time network to predict horizontal displacement errors based on outputs from the existing inertial navigation system and high-precision accelerometers.

The inputs to the displacement error prediction network include the three-dimensional gyroscope outputs from the original IMU, the three-dimensional accelerometer outputs (*m* accelerometers) from the original inertial navigation system, the three-dimensional high-precision accelerometer outputs (*k* accelerometers), and time information. These data are pre-integrated before being fed into the network, which then predicts displacement errors, outputting predictions over the selected pre-integration time window. The network uses the difference between the GPS-measured displacement and the displacement obtained from the Kalman filter-based inertial positioning algorithm as learning labels. Thus, the predicted errors belong to the low-frequency category relative to the original displacement errors. The network focuses on the variations in horizontal displacement errors, which is a critical factor in navigation accuracy.

To effectively utilize the temporal features of IMU samples, a time window of size k is used for data preparation through pre-integration, which is a technique initially proposed by Lupton [19] and widely used in inertial-vision positioning. In traditional inertial navigation calculations, all poses associated with a particular moment need recalculating when corrected by other sensor data, significantly increasing computational load. Pre-integration addresses this issue by estimating high-frequency IMU data in advance, allowing for prior estimations without initial state knowledge and requiring only bias correction for the pre-integrated frame when correction information is available, thus substantially reducing computation. The network predicts low-frequency displacement errors, and through pre-integration, it significantly reduces computation, enhances learning efficiency, and captures the nonlinear relationship between time information and errors.

Before entering the network, data pass through an embedding layer, where pre-integrated data undergo feature representation, then positional encodings provided by a trainable neural network are added and fed into both the encoder and decoder. Subsequently, the encoder, comprising N identical layer stacks, maps the input sequence into a continuous representation, capturing spatial information in the data via multi-head self-attention, with each block’s output serving as the next block’s input. The decoder also consists of N identical layer stacks, where masked self-attention is first executed to delve into the complex nonlinear relationship between displacement errors and time, ensuring that each timestamp t’s output depends only on data from before t, thus focusing on temporal dependencies. Multi-head attention combines spatiotemporal information into a single vector representation, followed by a fully connected feedforward network, with normalization processes included. Finally, a dense layer regresses the displacement error values from the network’s output. The learning rate is 0.0001, with 40 training epochs, using the ‘Adam’ optimizer. L2 regularization is employed within the network to prevent overfitting during training. The loss function for the displacement network is defined as follows:

(28) $$L_{E}(Error_{i},{\overset{\wedge }{Error}}_{i})=\frac{1}{2}{\left(Error_{i}-{\overset{\wedge }{Error}}_{i}\right)}^{T}{\sum \left(Error_{i}\right)}^{-1}{\left(Error_{i}-{\overset{\wedge }{Error}}_{i}\right)}^{T}+\frac{1}{2}\ln\left|{\sum \left(Error_{i}\right)}^{-1}\right|$$

## 4. Experimental Design and Implementation

### 4.1. Experiment Introduction

**Figure 5:**
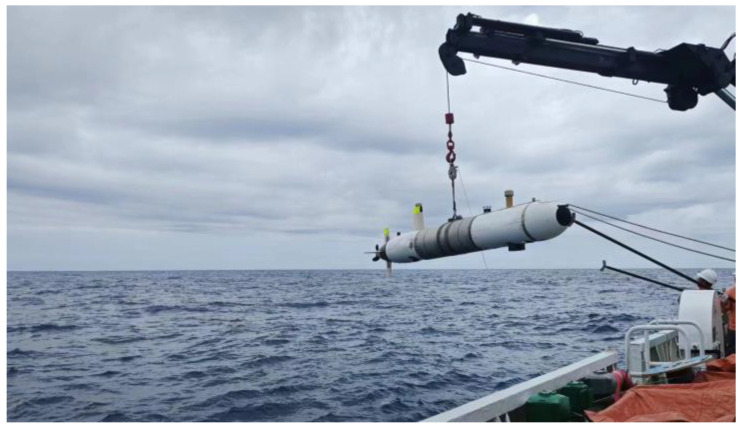
AUV diagram for experiment.

To validate the effectiveness of the methods proposed in this paper, the research team conducted sea trials near the Zhoushan area of Zhejiang, China, on 10 October 2023. The Redundant high-precision accelerometer-based inertial navigation system discussed in this document was deployed on an Autonomous Underwater Vehicle (AUV) platform, as illustrated in Figure 5.

The AUV platform comprises the AUV body, with the original inertial measurement unit equipped with quartz flexure accelerometer technology, additional high-precision accelerometers, an on-site data collection and control processor, a depth gauge, a satellite receiver, a Doppler velocimeter, a power system, a propulsion system, and an underwater communication system. The AUV platform was provided by DEEPINFAR Company, Tianjin, China. The inertial navigation equipment and high-precision accelerometers were provided by Central South University, Changsha, China. The software used in our experiments was Python, version 3.7. The propulsion system, which includes propellers, control units, and rudders, collaborates with the power system to propel the AUV. The underwater communication system ensures stable information exchange with the mother ship during experiments.

For our experiments, we utilized the original IMU and additional high-precision accelerometers for validating the final positioning solution, which meets the criteria for a pure inertial navigation experiment. The original IMU utilizes a fiber-optic gyro-based system with its built-in accelerometers also being quartz flexure accelerometers. While higher precision and smaller-sized optomechanical accelerometers and atomic interferometers exist and can offer several orders of magnitude greater precision than quartz flexure accelerometers, they are mostly still in the laboratory stage and have not become mature industrial products. Hence, we chose to continue using quartz flexure accelerometers for this research.

Specific parameters of the original IMU and the additional high-precision accelerometers are presented in Table 1 and Table 2, respectively. To evaluate the navigation performance of the proposed method, establishing a ground truth, such as a GPS position, is crucial. Before the actual navigation tests begin, the primary role of GPS is to provide training labels for the deep learning network. We used the dynamic GPS data as the truth value for the network to learn from in these preliminary stages. However, once the actual navigation tests commenced, GPS data were no longer involved in the navigation positioning process, adhering to the principle that “GPS data are used only before the real usage of the navigation system and do not receive any additional learning afterwards.” During this phase, GPS data served as a reference to validate the effectiveness of the proposed method in real-world experiments. This setup ensured that while the AUV could not acquire GPS positions underwater, the pre-test GPS data collection while cruising on the water surface offered a valuable baseline for comparing the effectiveness of the proposed navigation system against the actual GPS-obtained trajectories.

We anticipate that the displacement error prediction network will learn the characteristics of errors changing over time after the startup of the original IMU and additional high-precision accelerometer. Since the error factors of the devices change with each power cycle, continuous data collection for training and testing is performed after powering up the devices. Initially, data for training the displacement error prediction network are collected, documenting the AUV’s motion from acceleration startup to steady cruising and finally deceleration, with a total duration of 30 min, including various maneuvers, such as sharp turns and speed changes.

Subsequently, two sets of actual navigation tests, referred to as “Experiment 1” and “Experiment 2”, were conducted without power interruption and tailored to real-world scenarios. In Experiment 1, the AUV navigated at approximately 5 knots, covering a total distance of 48,360 m over 5 h. In Experiment 2, the AUV traveled at about 2 knots, covering 13,854 m over 4 h. Throughout these tests, data on acceleration, angular velocity, attitude angle, GPS positioning, and true heading were collected. The sampling rates for the IMU’s acceleration and angular velocity were 1000 Hz, with GPS data sampled at 1 Hz. Data files were saved every hour, with each data stream timestamped based on the system reading time for subsequent processing.

### 4.2. Evaluation Metrics

To further quantify and assess the performance of the method proposed in this paper compared to other methods, the Root Mean Squared Error (*RMSE*) was employed as the primary metric for evaluating the accuracy of pure inertial positioning in this experiment [20]. *RMSE* is defined as follows:

(29) $$RMSE=\sqrt{\frac{1}{m}\sum_{k}^{m}\left\|E_{t}(x_{k},{\overset{\wedge }{x}}_{k})\right\|}$$

where m represents the number of data points, k denotes the stamp, $E_{t}(x_{k},{\overset{\wedge }{x}}_{k})$ represents the ground truth position of the ground truth trajectory (i.e., $x_{k}$), and ${\overset{\wedge }{x}}_{k}$ represents the estimated position in the corresponding predicted path. Therefore, the following criteria were adopted as performance evaluation standards:

Relative Trajectory Error (RTE) is defined as the average *RMSE* over a fixed time interval. Due to the extended range of our test, we opted to prolong the evaluation interval to 30 s in contrast to standard ground navigation. For sequences shorter than 30 s, we computed the positional error at the last frame and adjusted the scale proportionally.

Additionally, given that many looping movements occur during the navigation, where error values may exhibit significant changes, it is essential to present the global Absolute Trajectory Error to assess error performance comprehensively.

### 4.3. Overall Performance

**Figure 6:**
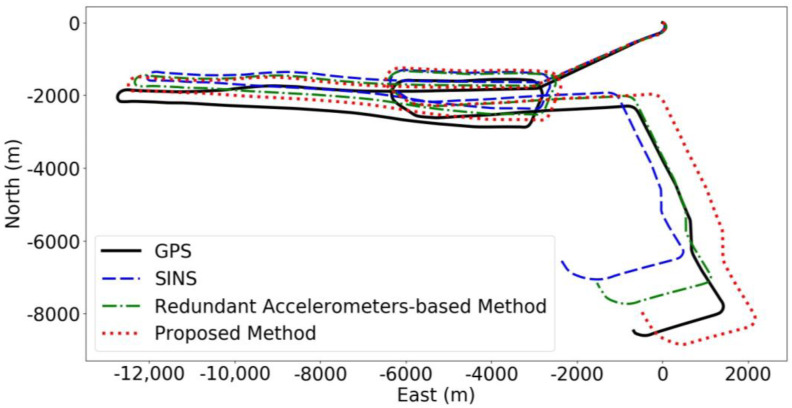
Full-course predicted trajectory diagram for Experiment 1.

In our experiment, we compared the performance of several methods to demonstrate the effectiveness of our proposed approach under long-distance conditions in pure inertial navigation. Specifically, we contrasted the “proposed method” with the “redundant accelerometers-based method” and the standard SINS navigation algorithm. The “redundant accelerometers-based method” refers to a configuration where high-precision accelerometers are added to the inertial navigation system but without the integration of our deep learning algorithm. This setup is intended to assess the impact of incorporating high-precision accelerometers alone on navigation accuracy. On the other hand, the “proposed method” employs not only the high-precision accelerometers but also integrates our specifically designed deep learning algorithm to enhance navigation precision and reduce errors. This dual approach leverages the advanced sensor hardware’s capabilities while also utilizing sophisticated machine-learning techniques to interpret and correct sensor data more effectively. Figure 6 shows the predicted trajectories for each method during the first group of navigation tests.

The comparative results from our experiments, as illustrated in Figure 6, reveal that the integration of high-precision accelerometers significantly enhances navigation accuracy beyond the capabilities of standard SINS. The method we designated as the “redundant accelerometers-based method” utilizes these advanced accelerometers to improve accuracy but does not incorporate our deep learning algorithm. On the other hand, our “proposed method”, which combines high-precision accelerometers with a deep learning algorithm, demonstrates a substantial further enhancement in reducing navigational errors and elevating overall system reliability and performance. This fusion of cutting-edge hardware with sophisticated algorithms markedly optimizes navigation accuracy. During the initial series of tests, conducted under comprehensive course conditions and depicted in Figure 6, our proposed method showcased superior performance, achieving the lowest final error at 574 m and a maximum error of 918 m. In stark contrast, the redundant accelerometers-based method resulted in both final and maximum course errors of 1585 m, whereas the baseline SINS, devoid of the additional high-precision accelerometers, recorded the highest discrepancies with final and maximum errors of 2644 m. This analysis highlights the critical advantage of integrating high-precision hardware with intelligent data processing algorithms to substantially enhance navigational precision.

**Figure 7:**
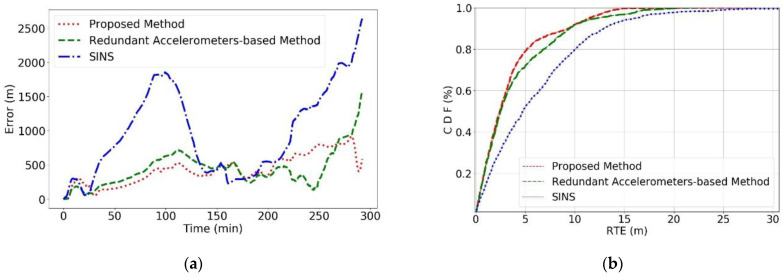
Error comparison of each method in Experiment 1. (**a**) Represents the global error and (**b**) represents the CDF of the RTE for the method.

Combining Figure 6 and Figure 7a, a global Absolute Trajectory Error analysis reveals that the proposed method consistently shows the lowest error values, significantly outperforming the other two methods, with the lowest final prediction error as well. There was a period between 140 and 200 min into the navigation where all three methods showed a sharp decline in error values, corresponding to the AUV’s circular motion phase in Figure 6, which does not fully reflect the actual error dynamics. Once the AUV completes the circular movement, a clear divergence in error values emerges, with SINS errors rapidly increasing. Figure 7b shows that the RTE of the proposed method is lower than the other methods, effectively reducing the RMSE values significantly compared to the original SINS method.

**Figure 8:**
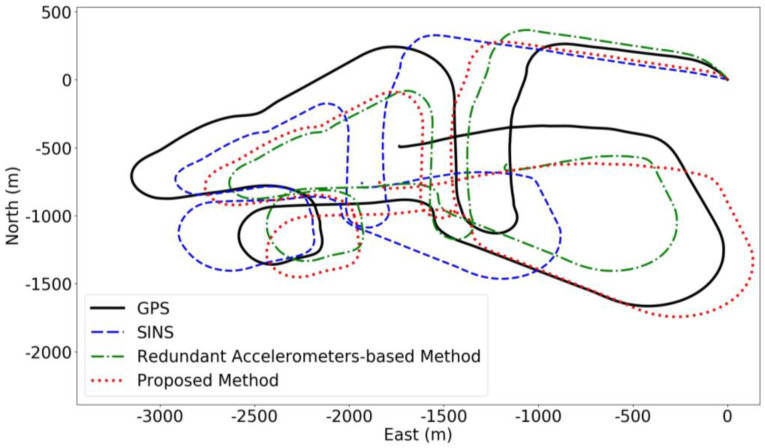
Full-course predicted trajectory diagram for Experiment 2.

As indicated in Figure 8, for the second set of experiments, the proposed method again showed the lowest final error and superior positioning performance throughout the course, with a final error of 251 m and a maximum error of 443 m. The redundant accelerometers-based method had a final error of 582 m and a maximum error of 607 m, while the SINS without the additional high-precision accelerometers had a final error of 1117 m and a maximum error of 1820 m. Compared to Experiment 1, the AUV’s speed was lower in Experiment 2, and it primarily performed irregular maneuvers, resulting in relatively lower final errors and a focus on the method’s robustness in complex maneuvering scenarios.

**Figure 9:**
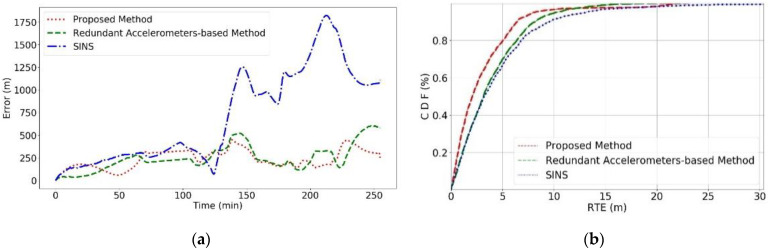
Error comparison of each method in Experiment 2. (**a**) Represents the global error and (**b**) represents the CDF of the RTE for the method.

Analyzing the global Absolute Trajectory Error in Figure 8 and Figure 9a, during the first half of the journey, all three methods show roughly the same error levels due to the AUV’s looping movements, making it challenging to reflect true error values. However, in the latter half, a clear increase in SINS errors is visible, while errors from the redundant accelerometers-based method momentarily drop to the lowest, likely due to coincidental trajectory overlap, which doesn’t truly represent its predictive accuracy. Overall, the proposed method still achieves the best predictive results. Figure 9b corroborates this, showing the proposed method’s RTE is less than that of the other methods, effectively reducing RMSE values. Moreover, the proposed method demonstrates higher predictive accuracy and robustness, maintaining reliable predictions globally.

The two experiments indicate that the method presented in this paper can effectively harness additional acceleration information provided by redundant high-precision accelerometers, enhancing the predictive accuracy of the inertial navigation system without altering its original structure. By introducing a displacement error prediction network based on redundant high-precision accelerometers, the system can better extract vehicular displacement error values from acceleration data, thereby inversely correcting the prediction results. Additionally, leveraging a Transformer-based displacement error prediction network to explore the time-varying nonlinear error characteristics inherent in inertial devices and motion processes significantly improves prediction accuracy. 

## 5. Conclusions

In this paper, we introduced an inertial navigation system structure that is augmented by redundant high-precision accelerometers and a sophisticated displacement error prediction network. By establishing a device-level redundant architecture that incorporates these high-precision accelerometers and harnessing a Transformer-based displacement error prediction network, our methodology aims to intricately predict and correct the nonlinear time-variant errors emanating from inertial navigation devices and vehicular motions, thereby aiming to enhance navigation accuracy. The validation of our approach through real-world maritime trials offers promising indications. While the results tentatively affirm the method’s capability to utilize redundant accelerometer data for refining navigation precision more effectively than conventional redundant systems, these findings underscore the necessity of cautious interpretation and further validation under a broader spectrum of experimental conditions.

In response to constructive feedback, we acknowledge the potential value of examining the proposed neural network independently, without integrating high-precision accelerometers, to isolate and evaluate the network’s intrinsic error correction capabilities. Such an investigation would provide a clearer understanding of the network’s standalone performance and its contribution to the system’s overall accuracy.

Future research extends beyond the scope of sea-level experiments to assess the displacement error prediction network’s performance in three-dimensional navigation, exploring the zenith-axis potential of high-precision accelerometers to furnish additional data, such as local acceleration and the complex nonlinear relationships between zenith acceleration changes and attitude adjustments. Moreover, forthcoming studies will examine the performance of the error inversion network when used independently, without the aid of high-precision accelerometers. This approach will allow us to specifically assess the network’s effectiveness and contribute to a more focused comparative analysis of the system’s performance. Ultimately, our ongoing research endeavors will continue to explore the boundaries of precision enhancement capabilities provided by redundant accelerometers in inertial navigation systems, venturing into new research avenues within the domain of inertial navigation.

## Figures and Tables

**Table 1:** High-precision accelerometer key performance parameters.

Parameter Item	Parameter Value
Accelerometer Bias Stability/μg	1
Velocity Random Walk/[μg/√Hz]	0.1
Accelerometer Scale Factor Nonlinearity/ppm	2

**Table 2:** Key performance parameters of the original fiber optic inertial navigation system.

Parameter Item	Parameter Value
Gyroscope Bias Stability/[(°)/h]	0.01
Angular Random Walk/[(°)/√h]	0.001
Gyroscope Scale Factor Nonlinearity/ppm	20
Accelerometer Bias Stability/μg	<10
Accelerometer Velocity Random Walk/[μg/√Hz]	1
Accelerometer Scale Factor Nonlinearity/ppm	20

## Data Availability

Data available on request due to restrictions. Due to the sensitive nature of our institution, the dataset from this study is not openly available. However, if access to the data is required, interested parties may contact the corresponding author. Upon request, and following a review by our institution, the data may be shared under specific conditions. This ensures compliance with our institutional policies while facilitating potential collaboration and further research.
